# Methyl parathion detection in vegetables and fruits using silver@graphene nanoribbons nanocomposite modified screen printed electrode

**DOI:** 10.1038/srep46471

**Published:** 2017-04-20

**Authors:** Mani Govindasamy, Veerappan Mani, Shen-Ming Chen, Tse-Wei Chen, Ashok Kumar Sundramoorthy

**Affiliations:** 1 Department of Chemical Engineering and Biotechnology, National Taipei University of Technology, Taipei, Taiwan 106 ROC; 2 Graduate Institute of Biomedical and Biochemical Engineering, National Taipei University of Technology, Taipei, Taiwan 106 ROC; 3 SRM Research Institute, Department of Chemistry, SRM University, Kattankulathur, 603203, Chennai, Tamilnadu, India

## Abstract

We have developed a sensitive electrochemical sensor for Organophosphorus pesticide methyl parathion (MP) using silver particles supported graphene nanoribbons (Ag@GNRs). The Ag@GNRs nanocomposite was prepared through facile wet chemical strategy and characterized by TEM, EDX, XRD, Raman, UV-visible, electrochemical and impedance spectroscopies. The Ag@GNRs film modified screen printed carbon electrode (SPCE) delivers excellent electrocatalytic ability to the reduction of MP. The Ag@GNRs/SPCE detects sub-nanomolar concentrations of MP with excellent selectivity. The synergic effects between special electrocatalytic ability of Ag and excellent physicochemical properties of GNRs (large surface area, high conductivity, high area-normalized edge-plane structures and abundant catalytic sites) make the composite highly suitable for MP sensing. Most importantly, the method is successfully demonstrated in vegetables and fruits which revealed its potential real-time applicability in food analysis.

Pesticides, such as Organophosphorus are widely used in agriculture to protect agricultural crops from damages caused by insects in order to increase food production^1^,^2^. However, the increasing use of pesticides in agriculture has generated numerous concerns in food safety. More than 70% of pesticides used all over the world are OP pesticides and its derivatives^3^,^4^. Methyl parathion (MP), a Organophosphorus (OP) pesticides is extensively used in agricultural crops to increase food production^5^,^6^. But, the presence of lethal amounts of residual MP in food products is a big concern for food safety^7^. The residues of MP have high environmental persistent in food and water which impose big threats^8^,^9^. Hence, there is urgent need to develop rapid and robust detection methods for the detection of MP in food samples^10^. Despite the excellent accuracy of gas chromatography (GC), high performance liquid chromatography (HPLC) and gas chromatography-mass spectrometry (GC-MS) for MP detection, they are laborious and limited in centralized laboratories^11^. The biological detection methods (immunoassays^12^ and acetylcholine esterase^7^) coupled with optical/electrochemistry read-outs have shown comparable performance; however, they involve extensive sample handing, require prolonged analysis time and pre-treatment steps^3^. Enzymatic biosensors based on Organophosphorus hydrolase have been developed in which the enzyme catalyzes the hydrolysis of MP and produce *p*-nitro phenol which is detected via colorimetry/electrochemistry^8^,^13^; however, enzyme instability and denaturation during immobilization and storage period make this method not suitable for on-line applications. On the other hand, electroanalytical methods are simple, fast, low-cost, portable, and easy-to-use. Several electroanalytical sensors have been reported for MP sensing based on different chemical modifiers which includes, electrochemically reduced graphene oxide^14^, nanosilver/nafion composite^1^, zirconium oxide nanoparticles^4^, zirconia/ordered macroporous polyaniline^15^, molecularly imprinted polymer–ionic liquid–graphene composite^16^, Au nanoparticles/nafion^17^, ordered mesoporous carbon^18^, and carbon nanotubes–poly(acrylamide) nanocomposite^19^. In recent times, graphene is widely popular electrode materials in electroanalysis and numerous graphene based nanomaterials are enjoying widespread popularity in MP sensor^5^,^16^,^20^,^21^. More recently, graphene nanoribbons (GNRs), strips of graphene nanosheets having confined width in nanometers (<50 nm) are emerging as another interesting carbonaceous nanomaterial^22^,^23^,^24^,^25^. Compared with the inert chemical surface of pristine graphene nanosheets, GNRs have significantly higher rich edge chemistry, abundant functional groups, higher area-normalized edge-plane structures and more active sites which can be useful for electrocatalysis^26^,^27^. However, GNRs based materials are not widely studied as much as graphene in sensing applications^26^,^28^,^29^. On the other hand, we understood from the literature study that silver particles (Ag) have special electrocatalytic ability towards MP and it significantly accelerates the electrocatalysis reaction^30^. Recently, D.J Davis *et al*., reported the preparation of Ag@GNRs via chemical unzipping of K-intercalated MWCNTs by reaction with Na/K alloy, Ag(O_2_CCH_3_) and then CH_3_OH for oxygen reduction reaction (ORR), but the synthetical procedure is dangerous since it uses Na/K alloy^31^. Recently, J.E.S. Fonsaca *et al*., reported the preparation of cube-like Ag-nanoparticle on cysteamine functionalized GNRs using sodium borohydride as reductant and utilized it for detecting molecules via surface enhanced Raman spectroscopy (SERS)^32^.

Herein, we are preparing Ag@GNRs nanocomposite via simple wet-chemical method for the sensitive determination of methyl parathion residues in vegetables and fruits by considering the significance MP detection in food safety. First, graphene oxide nanoribbons (GONRs) were prepared through acid treatment and next it was reduced to GNRs along with the simultaneous decoration of Ag particles onto the nanosheets. We have demonstrated the Ag@GNRs modified screen printed carbon electrode (SPCE) as suitable electrode for MP detection in fruit and vegetables. Generally, graphene and metal nanoparticles have excellent synergic effect in their composite and here we can expect similar synergic effects in Ag@GNRs. We have adopted screen printed carbon electrodes (SPCE) to prepare working electrode because of its low-cost, easy fabrication, flexibility, and reproducibility. Most importantly, the developed nanocomposite is successfully verified in the real-time analysis of MP in food samples such as vegetables (cabbage and green beans) and fruits (strawberry and nectarine fruit).

## Results and Discussions

### Characterizations

**Figure 1:**
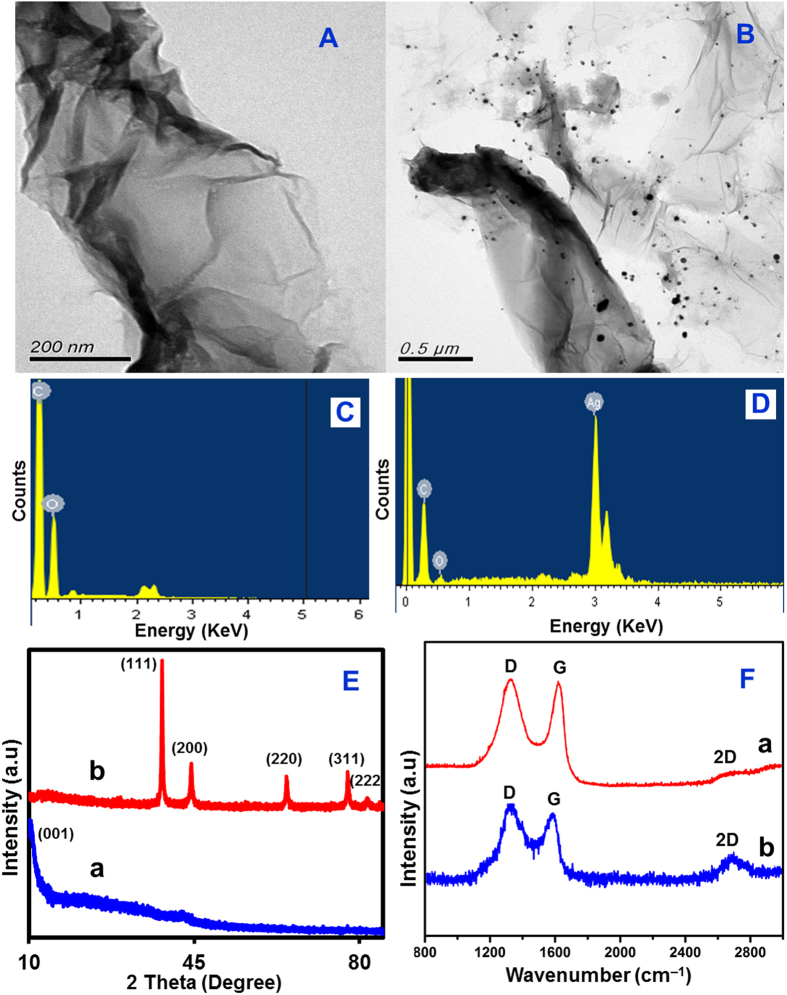
TEM images of GONR (**A**) and Ag@GNRs (**B**). EDX spectra of GONR (**C**) and Ag@GNRs (**D**). XRD (**E**) and Raman spectra (**F**) of GONRs (a) and Ag@GNRs (b).

The TEM images of GONRs revealed the unzipped outer walls of nanotubes as sheets of nanoribbon (Fig. 1A). The nanoribbon consists of different layers and the sheets thicknesses are about 1 nm. During acid treatment of MWCNTs, the C = C bond underwent chemical oxidization and led to the formation of oxygen functionalities. These oxygen functionalities generate large negative inductive effect, as a result the tubes experiences repulsive force which led to longitudinally unzipping^28^,^33^. The EDX data of GONRs (Fig. 1C) displays the presences of expected elements, carbon (C) and oxygen (O) with weight% of 61.49 and 38.51 and atomic% of 69.84 and 30.16, respectively. The TEM image of Ag@GNRs displays the distribution of Ag nanoparticles and particles with an average particle size varies in nanometers on the thin and smooth surface of GNRs sheets (Fig. 1B) and this morphology is similar to the previously reported graphene/Ag based composites^34^. The EDX spectrum of Ag@GNRs (Fig. 1D) featured with the presence of C, O and Ag elements with weight% of 20.21, 5.09 and 74.70, and atomic% of 59.56, 12.50 and 27.93, respectively and hence the EDX results revealing the high loading of Ag. Interestingly, the amount of oxygen is considerably reduced from GONRs (38.51%) to Ag@GNRs (5.09%) signifying the reduction of GONRs to GNRs along with the formation of Ag particles.

Figure 1E displays the XRD patterns of GONRs (a) and Ag@GNRs (b). The XRD pattern of GONRs features with characteristic peak at 10.1° related to the large interlayer d-spacing of 8.02 Å. The observation of this peak is strong evidence for the formation of GONRs. This peak is disappeared in the XRD pattern of Ag@GNRs suggesting the reduction of GONRs to GNRs. In addition, a set of five diffraction peaks at 38.15, 44.19, 64.41, 77.32 and 81.94° are observed which are manifested to (111), (200), (220), (311) and (222) reflections of face-centered cubic structure of silver (JCPDS, File No. 4-0783). The Raman spectra of GONRs (a) and Ag@GNRs (b) are given in Fig. 1F.

The Raman spectrum of GONRs featured with sharp D band at 1340 cm^−1^ (related to defects) and G band at 1587 cm^−1^ (originates from the stretching of in-plane sp^2^ atoms). The level of disorder in the graphene materials can be elucidated by analyzing the ratio of peak intensities *I*_D_/*I*_G_. The value of *I*_D_/*I*_G_ for GONRs was 0.98 higher than that for pristine MWNTs^35^ and this result clearly revealed the increased defect density in GONR which can be accounted for the generated large numbers of edge sites. The value of *I*_D_/*I*_G_ was further increased to 1.08 for Ag@GNRs and this result is accounted for the formation of graphitic domains that are smaller in average size, but more numerous than in GONRs^29^,^35^. The 2D band (a second-order overtone of the D band) was observed with very low intensity in the spectrum of GONRs as a result of extensive oxidation which is consistent with previous reports^23^,^35^. However, the intensity of 2D band was considerably increased in the spectrum of Ag@GNRs which indicates the partial restoration of graphitic network during reduction process (i.e., GONRs to GNRs).

**Figure 2:**
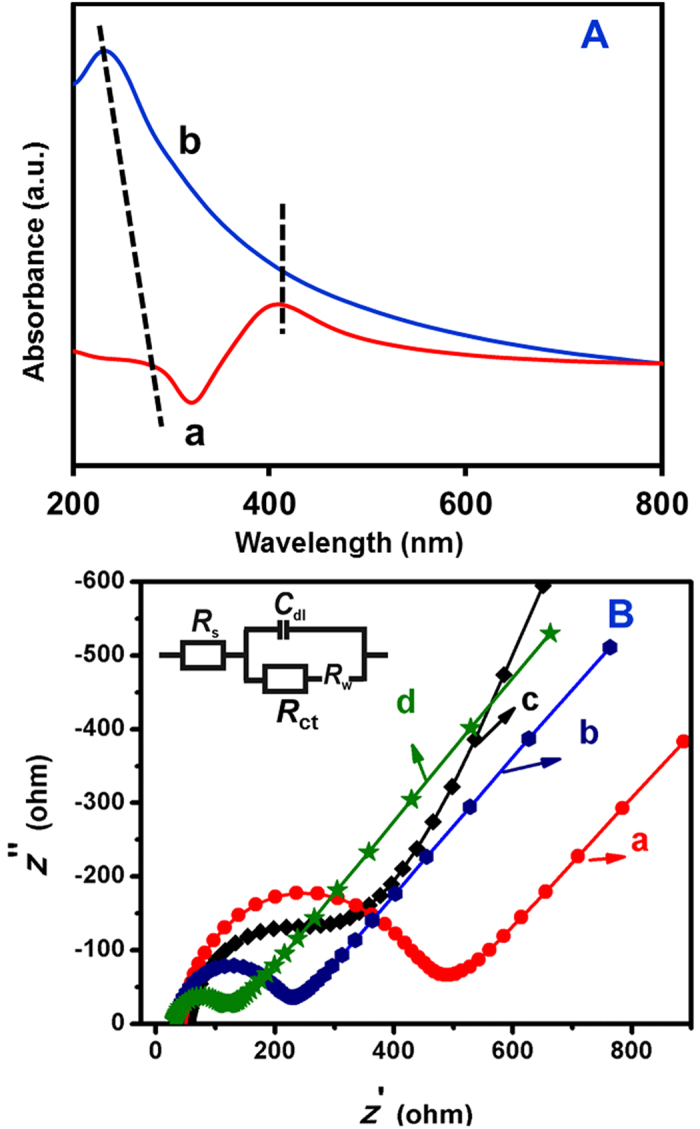
UV–visible (**A**) spectra GONRs (a) and Ag@GNRs (b). EIS curves of unmodified SPCE (a) Ag/SPCE (b), GONRs/SPCE (c) and Ag@GNRs/SPCE (d) in 0.1 M KCl containing 5 mM Fe(CN)_6_^3−/4−^. Amplitude: 5 mV, Frequency: 0.1 Hz to 100 kHz. Inset: Randles equivalent circuit model has been used to fit the experimental data in which, *R*_s_, *R*_ct_ *C*_dl_ and *Z*_w_ are representing electrolyte resistance, charge transfer resistance, double layer capacitance and Warburg impedance, respectively.

Furthermore, GONR and Ag@GNRs are characterized using UV-Visible spectra (Fig. 2A). Generally, highly conjugated graphene-like material will have a higher *λ*_max_ while material with a disrupted π–network and greater number of sp^3^ carbons will have a lower *λ*_max_^33^. Here, *λ*_max_ observed for GNRs–Ag (300 nm, curve b) is significantly higher than the GONR (248 nm, curve a), which means the disrupted π–network of GONR is considerably restored in the composite. Besides, the *λ*_max_ is red shifted from GONR to Ag@GNRs which indicates the possible electronic interaction between graphene surface and Ag particles. In addition, a new absorption peak corresponding to the Ag particles is observed in the spectrum of Ag@GNRs which is an additional evidence for the successful formation of Ag particles. Figure 2B displays the EIS curves obtained at unmodified SPCE (a), Ag/SPCE (b), GNRs/SPCE (c) and Ag@GNRs/SPCE (d) using Fe(CN)_6_^3−/4−^ as the redox mediator. Randles equivalent circuit model has been used to fit the experimental data (inset to Fig. 2B), in which, *R*_s_, *R*_ct_ *C*_dl_ and *Z*_w_ are representing electrolyte resistance, charge transfer resistance, double layer capacitance and Warburg impedance, respectively. The *R*_ct_ values obtained for unmodified SPCE, Ag/SPCE, GNRs/SPCE and Ag@GNRs/SPCE are 460.1 (±1.1), 189.0 (±1.5), 223.6 (±1.4), and 71.4 (±1.7) Ω, respectively. The *R*_ct_ value obtained at Ag@GNRs/SPCE is the lowest which indicating that the charge transfer resistance at the interface of Ag@GNRs/SPCE and electrolyte is minimum compared with other electrodes. In other words, Ag@GNRs has high electrical conductivity over control electrodes and this behaviour is highly useful in electrochemical sensing applications.

#### Electrocatalysis of methyl parathion

**Figure 3:**
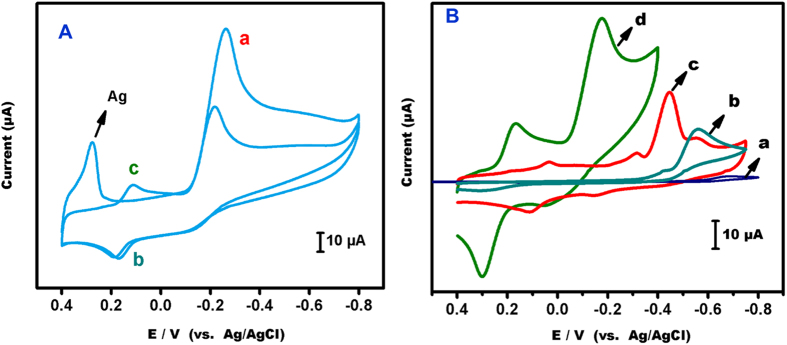
(**A**) Cyclic voltammograms of Ag@GNRs/SPCE in phosphate buffer (pH 7) containing 2 μM MP. Scan rate = 50 mVs^−1^. (**B**) Cyclic voltammograms obtained at (a) bare SPCE, (b) GONRs/SPCE, (c) Ag/SPCE (d), Ag@GNRs/SPCE in phosphate buffer (pH 7.0) containing 2 μM MP.

Electrocatalytic ability of the Ag@GNRs/SPCE towards electrocatalysis of MP (2 μM) reduction is investigated in phosphate buffer (pH 7) (Fig. 3A). The forward segment of first cycle displays a sharp cathodic peak at −0.18 V which is manifested to the reduction of NO_2_–MP to NHOH–MP (Eq. 1). An oxidation peak is observed at +0.18 V in the backward segment, which is related to the oxidation of NHOH–MP to NO–MP and this reaction is reversible and hence corresponding reduction is also observed at +0.15 V during the second cyclic run. This type of electrocatalytic behaviour is consistent with the previous reports^14^. Moreover, we have observed a sharp oxidation peak at +0.29 V which is due to the oxidation of Ag.


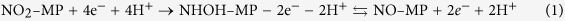


**Figure 4:**
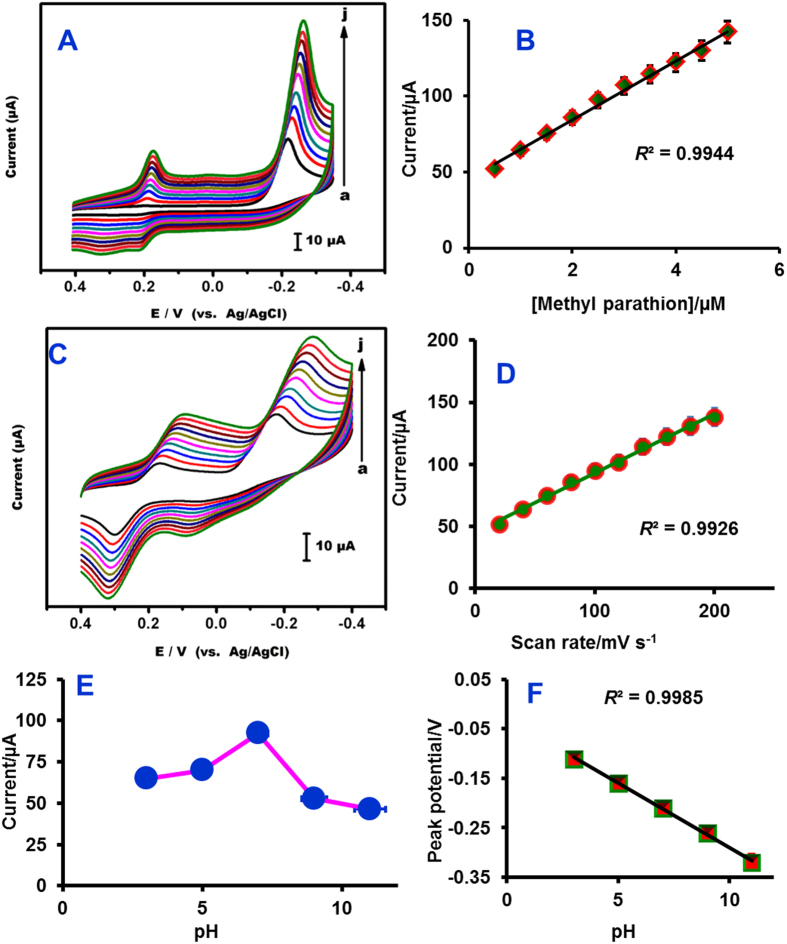
(**A**) Cyclic voltammograms of MP at Ag@GNRs/SPCE towards different concentrations of MP (a = 0.5, b = 1.0, c = 1.5, d = 2.0, e = 2.5, f = 3.0, g = 3.5, h = 4.0, i = 4.5 and j = 5.0 μM). Scan rate = 50 mVs^−1^. (**B**) Calibration plot of 4 A; Current (μA) vs. [methyl parathion]/μM. (**C**) Cyclic voltammograms of Ag@GNRs/SPCE in phosphate buffer (pH 7) containing 2 μM MP at different scan rates (20 to 200 mV s^−1^). (**D**) Scan rate (mV s^−1^) vs. peak currents (μA). (**E**) Dependence of cathodic peak current with respect to pH. The voltammograms are performed using Ag@GNRs/SPCE in phosphate buffer (pH 7) containing 2 μM MP for different pH studies (**F**) Dependence of cathodic peak potential with pH.

In this study, we have focused on the reduction peak of MP (NO_2_–MP to NHOH–MP), because this reduction process is more suitable for sensor applications. Accordingly, the MP reduction process at Ag@GNRs/SPCE is monitored and compared with control electrodes (Fig. 3B). In comparison with control electrodes (bare SPCE, GNRs/SPCE and Ag/SPCE), the Ag@GNRs/SPCE is shown significantly enhanced reduction peak current. The observed overpotentials at bare SPCE, GNRs/SPCE, Ag/SPCE and Ag@GNRs/SPCE are −0.70 V, −0.58 V, −0.42 and −0.18 V, respectively. The overpotential at Ag@GNRs/SPCE is 520 mV, 400 mV and 240 mV lower than the bare SPCE, GNRs/SPCE and Ag/SPCE. The drastic shift in the overpotential at Ag@GNRs/SPCE is due to the electrocatalytic activity of Ag which greatly accelerates and catalyzes the reduction process^1^. The synergic effect of GNRs and Ag is obvious because control electrodes have poor electrocatalytic ability, while Ag@GNRs has significantly improved electrocatalytic performance. The electrocatalytic activity of GNRs is mainly due to the absorption of MP through π stacking interaction between benzene ring of MP and aromatic moieties of GNRs. Besides, there are also other types of interactions such as electrostatic and hydrogen bonding between MP and Ag@GNRs. The contribution of defect sites and oxygen functionalities created on the SPCE via pre-anodization is also significantly factor^36^. Also, the reduction peak current is linearly increased with respect to MP concentration (Fig. 4A) and the plot between reduction peak current and concentration of MP is exhibited good linearity (Fig. 4B).

#### Electrode kinetics and effect of pH

Next, the effect of scan rate on the reduction of MP was studied by applying different scan rates from 20 to 200 mVs^−1^ (Fig. 4C). The reduction peak current of MP is linearly increases as the scan rate increases. The plot between peak current and scan rate follows linear behaviour which is a characteristic of surface-confined diffusion controlled electrocatalytic process (Fig. 4D). The influence of buffer pH on the electrocatalysis of MP was investigated (Fig. 4E). As the pH of supporting electrolyte changes, both peak current and peak potential of the MP reduction are changed. The peak current increases as pH increases from 3 to 7 and reached maxima at pH 7 and it decreases on further increase in pH. The plot between different pH and peak potential is exhibits good linearity (Fig. 4F).

#### Determination of methyl parathion

**Figure 5:**
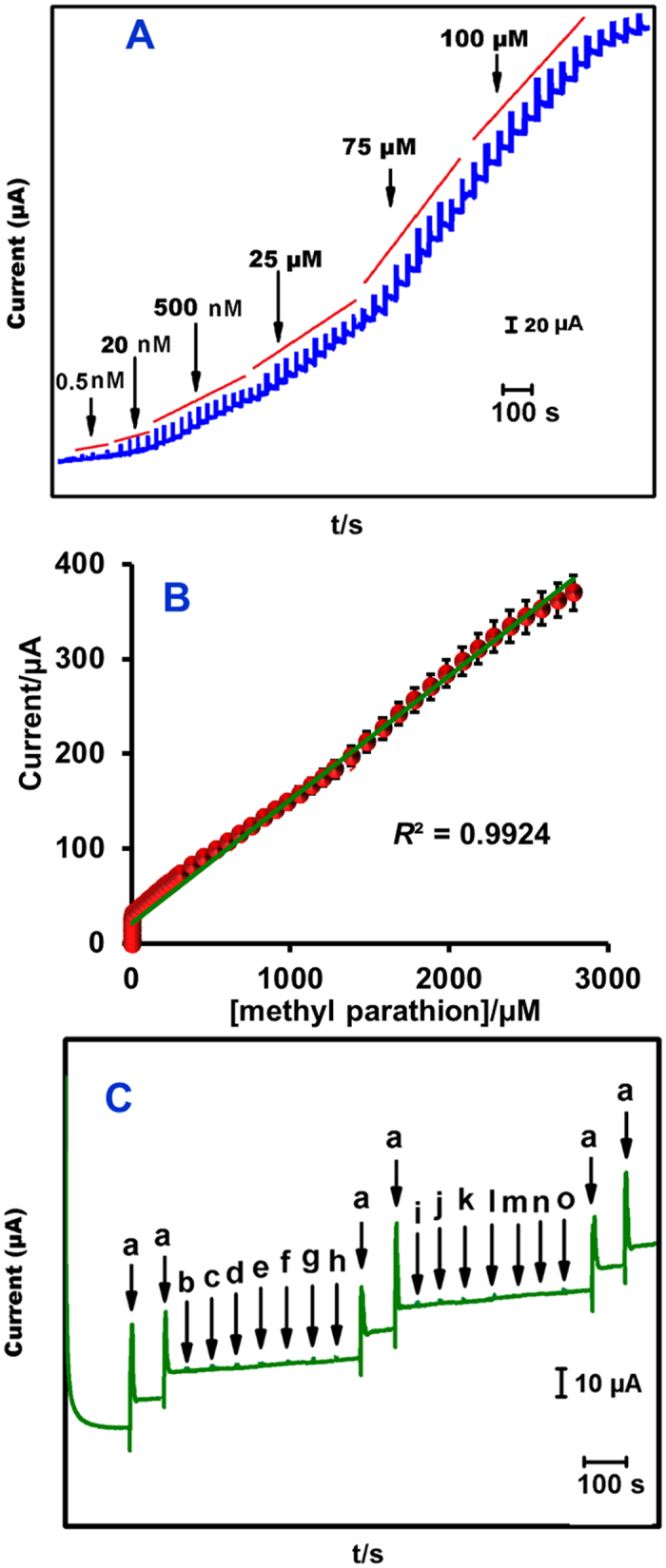
(**A**) Amperometric responses of Ag@GNRs/SPCE towards each sequential additions of MP into phosphate buffer (pH 7). The rotation speed = 1500 RPM and electrode potential = −0.18 V. (**B**) [methyl parathion]/μM vs. current (μA). (**C**) Selectivity study: Amperometric response of Ag@GNRs/SPCE for 50 μM MP (a), Ca^2+^ (b), Cu^2+^ (c), Mn^2+^ (d), Ba^2+^ (e), Ni^2+^ (f), Zn^2+^ (g), NO_3_^−^ (h), Malathion (i), 4–Nitrophenol (j), 4–Nitrobenzene (k), 4–Aminophenol (l), 2–Nitro aniline (m), 4–Nitro aniline (n) and 4–acetamidophenol (o).

Figure 5A represents the amperometric curves obtained at Ag@GNRs film modified electrode for sequential additions of MP into phosphate buffer (pH 7). The applied potential was −0.18 V and the rotation speed was 1300 RPM. For each addition, a sharp increase in the amperometric current is observed and the response current reached 95% steady-state current within 5 s of MP injection. The concentration dependent linear plot displays good linearity (Fig. 5B). The working concentration range is 5 nM–2780 μM and the sensitivity is 0.5940 μAμM^−1^ cm^−2^. The detection limit is 0.5 nM. The important parameters of sensor, such as detection limit and linear range are compared with previously modified electrodes (Table 1)^1^,^16^,^17^,^18^,^19^,^37^,^38^,^39^,^40^,^41^. The analytical performance of the Ag@GNRs/SPCE is competitive to the previously reported MP sensors.

#### Selectivity

Selectivity of the Ag@GNRs/SPCE to detect MP in presence of possible interferents has been investigated (Fig. 5C). The tested interferents are Ca^2+^ (b), Cu^2+^ (c), Mn^2+^ (d), Ba^2+^ (e), Ni^2+^ (f), Zn^2+^ (g), NO_3_^−^ (h), 4-Acetaminophenol (i), 4-Nitrophenol (j), 4-Nirobenzene (k), 4-Aminophenol (l), 2-Nitro aniline (m), 4-Nitro aniline (n) and 4-acetamido phenol (o). As shown in the figure, the modified electrode has delivered excellent current response to MP, but it shows negligible responses to the tested other analytes. The possible reason for the selectivity is different interferents have different redox potentials and different adsorption sites on the surface of GNRs–Ag, while the adsorption of MP could be significantly higher than the other species. The applied potential is drastically lowered which is one of the important reasons for selectivity.

#### Stability, repeatability and reproducibility studies

In order to determine storage stability of the Ag@GNRs/SPCE, its electrocatalytic response to 100 nM MP was monitored every day. The electrode delivered consistent amperometric responses to MP during two weeks of storage period. About 92.03% of initial response current was retained after two weeks of its use which indicating the electrode’s good stability. Next, repeatability and reproducibility of the modified electrode are studied. The electrode exhibits appreciable repeatability with RSD of 4.51% for five repeatitive measurements performed using single modified electrode. Similarly, the electrode exhibits good reproducibility with RSD of 4.36% for five independent measurments perfomed in five independent modified electrodes.

#### Real sample analysis

**Figure 6:**
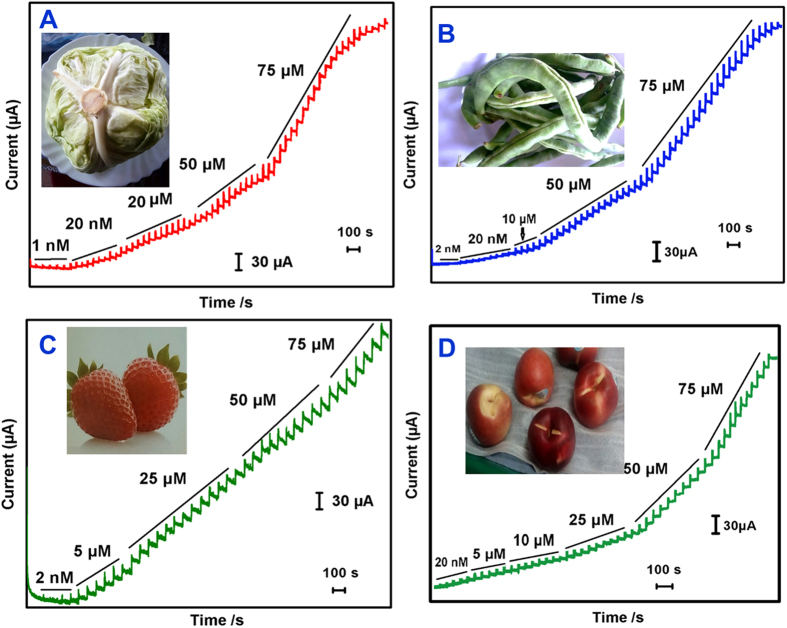
Real sample analysis: Amperometric responses of Ag@GNRs/SPCE for each sequential addition of real samples containing MP into continuously stirred phosphate buffer (pH 7). (**A**) cabbage, (**B**) Green beans, **C**) Strawberry and (**D**) nectarine fruit. Amperometric experiments are performed using Ag@GNRs/SPCE towards each sequential addition of real samples into phosphate buffer (pH 7). The rotation speed = 1500 RPM and electrode potential = −0.18 V.

**Figure 7:**
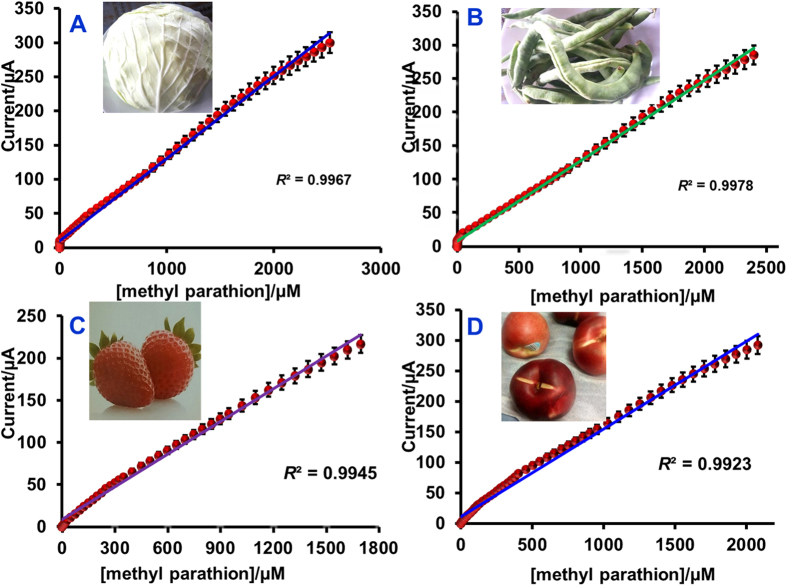
Calibration plots for real sample analysis; [methyl parathion] (μM) vs. Current (μA). (**A**) cabbage, (**B**) Green beans, **C**) Strawberry and (**D**) nectarine fruit.

Figure 6 displays the amperometric curves obtained for the determination of MP present in cabbage (A), Green beans (B), Strawberry (C) and nectarine fruit (D) using Ag@GNRs/SPCE. As shown in figure, well-defined amperometric responses are observed for the each addition of real samples in the supporting electrolyte. The response current reached 95% steady-state current within 5–7 s of the sample injection and the resulting amperogram are consistent with lab sample results. The concentration dependent linear calibration plots have shown good linearity as shown in Fig. 7 (A. cabbage, B. Green beans, C. Strawberry and D. nectarine fruit). The sensor parameters such as working range, sensitivity and detection limit obtained for the fruits and vegetables have been calculated. The linear range, LOD and sensitivity for detection of MP in cabbage samples are 2 nM–2525 μM, 1.0 nM, and 0.559 μAμM cm^−2^ respectively. For green beans sample, the linear range, LOD and sensitivity are 4 nM–2400 μM, 2.0 nM, and 0.569 μAμM cm^−2^ respectively. For strawberry sample, the linear range, LOD and sensitivity are 6 nM–1700 μM, 2.0 nM, and 0.611 μAμM cm^−2^ respectively. For Nectarine fruit sample, the linear range, LOD and sensitivity are 4 nM–2080 μM, 3.0 nM, and 0.683 μAμM cm^−2^ respectively.

## Conclusions

In summary, a highly sensitive methyl parathion sensor is developed for the determination of MP in cabbage, green beans, strawberry, and nectarine samples using Ag@GNRs/SPCE. The Ag@GNRs is successfully prepared through simple wet chemical method and its structure was confirmed by TEM, EDX, XRD, Raman, UV-visible and EIS techniques. The synergic combination of GNRs and Ag greatly reduced the overpotential and enhanced the sensitivity. The drastic reduction in overpotential offers the advantages of less energy requiremet for reduction process and eliminates interferences at high potential region. The modified electrode has excellent sensor performance and achieved low detection limit. The other advantages of the electrode are its reproducibility, sensitivity, selectivity, stability, repeatability, fast response time and low-cost fabrication. The advantages of SPCE technology in combination with excellent electrocatalytic attributes of Ag@GNRs make the composite highly suitable for electroanalytical applications and the composite is thus expected to open new opportunities for the pesticide sensing in food samples.

## Experimental

### Chemicals and Apparatus

MWCNTs (bundled >95%), siver nitrate (AgNO_3_), MP and all other reagents including solvents were purchased from Sigma-Aldrich and used as received. Electrochemical studies were performed in a conventional three electrode cell using modified SPCE as a working electrode (area 0.3 cm^2^), saturated Ag|AgCl (saturated KCl) as a reference electrode and Pt wire as a counter electrode. The SPCEs were purchased from Zensor R&D Co., Ltd., Taipei, Taiwan. Prior to each electrochemical experiment, the electrolyte solutions were deoxygenated with pre-purified nitrogen for 15 min unless otherwise specified. The supporting electrolyte used for the electrochemical studies was 0.1 M phosphate buffer (pH 7) prepared from sodium dihydrogen phosphate and disodium hydrogen phosphate. 0.1 M acetate buffer (for pH 3 and 5), 0.1 M phosphate buffer (for pH 7) and 0.1 M Tris-buffered saline (for pH 9) were prepared and used for different pH studies.

All the electrochemical measurements were carried out using CHI 1205 A electrochemical work station (CH Instruments, Inc., U.S.A) at ambient temperature. Surface morphological studies were carried out using Hitachi S-3000 H scanning electron microscope (SEM) and transmission electron microscope (TEM) (H-7600, Hitachi, Japan). Energy-dispersive X-ray (EDX) spectra were performed using Horiba Emax x-act (Sensor +24 V = 16 W, resolution at 5.9 keV). EIM6ex Zahner (Kronach, Germany) was used for electrochemical impedance spectroscopy (EIS) studies. X-ray diffraction (XRD) diffraction studies were carried out using XPERT-PRO (PANalytical B.V., The Netherlands) diffractometer (Cu Ka radiation, *k* = 1.54 Å).

### Preparation of graphene oxide nanoribbons

300 mg of MWCNTs were added to 80 mL H_2_SO_4_ and stirred for 1 h. Subsequently, 8 mL H_3_PO_4_ was added and the solution was stirred for another 20 min. Next, 2.5 g of KMnO_4_ was added and the whole mixture was heated at 65 °C for 2 h and finally cooled to room temperature. Afterwards, the reaction mixture was poured on 100 mL of ice containing 10 mL 30% H_2_O_2_. The obtained brown colored sediment was filtered and washed with 100 mL of water. Next, it was washed with 3× with HCl (20 vol%, 25 mL each), 2× with ethanol (20 mL each) and 2× with ether (20 mL each). Finally, the purified GONRs were vacuum dried for overnight at 80 °C. GONRs were redispersed in water (1 mg mL^−1^).

### Synthesis of Ag@GNRs nanocomposite

In a standard synthesis of Ag@GNRs nanocomposite, first 10 mg of GONRs was suspended in 30 mL of water via ultrasonication for 20 min. 175 mg AgNO_3_ dissolved in 20 mL water was added to the GONRs dispersion and continued ultrasonication for another 20 min. Next, the mixture was transferred to a round bottom flask and heated upto 90 °C with continuous stirring using magnetic stirrer. Subsequently, 1 g sodium citrate dissolved in 40 mL water was added and the whole reaction mixture was heated with refluxed at 90 °C for 24 h. The product Ag@GNRs nanocomposite isolated via centrifugation and further washing steps with copious amount of water and ethanol. Ag@GNRs was overnight dried at 80 °C and re-dispersed (1 mg mL^−1^) in water/ethanol mixture (60/40;v/v%) through ultrasonication for 30 min. As control, GNRs and Ag were prepared individually and redispersed in water (1 mg mL^−1^).

### Preparation of modified SPCE

First, the surface of SPCE was pre-cleaned by cycling between −1.0 V and 1.2 V, in 0.1 M phosphate buffer (pH 7). Next, the SPCE was preanodized by applying 2.0 V (vs. Ag/AgCl) constant potential for 300 s in 0.1 M phosphate buffer (pH 7)^34^. Then, 5 μl dispersion of Ag@GNRs is drop casted on the preanodized SPCE surface and dried at ambient conditions. As control, GNRs/SPCE and Ag/SPCE are prepared accordingly.

### Sampling procedure for real samples

Fresh cabbage, green beans, strawberry and nectarine fruit were purchased from local supermarket and washed with water. (1) To prepare cabbage sample solution, it was sliced and different concentrations of MP (stock solution) are sprayed onto these slices. About 10 g of cabbage slices were added to 20 mL phosphate buffer (pH 7) and blended using a blender. Next, the solution was centrifuged (3000 RPM) and the supernatant was used as stock solution and used for real sample analysis. (2) To prepare green beans, known concentrations of MP are sprayed onto the pieces green beans skin. After 1 h, the skin of the green beans were separated and sliced. About 10 g of green beans slices were added to 20 mL phosphate buffer (pH 7) and blended using a blender. Next, the solution was centrifuged (3000 RPM) and the supernatant was used as stock solution and used for real sample analysis. (3, 4) The strawberry and nectarine fruit samples were cut into pieces and known concentrations of MP are sprayed onto the pieces using a sprayer. After 2 h of air drying, the pieces of the fruits samples were peeled using a fruit peeler, and then the peel were further cut into smaller pieces. About 5 g of fruits peels are added to 20 mL of phosphate buffer (pH 7) and the mixture was shaken vigorously for 20 min using a stirrer. The centrifugate was collected as a stock solution and subsequently used for analysis.

## Additional Information

**How to cite this article**: Govindasamy, M. *et al*. Methyl parathion detection in vegetables and fruits using silver@graphene nanoribbons nanocomposite modified screen printed electrode. *Sci. Rep.* **7**, 46471; doi: 10.1038/srep46471 (2017).

**Publisher's note:** Springer Nature remains neutral with regard to jurisdictional claims in published maps and institutional affiliations.

## Figures and Tables

**Table 1:** Comparison of analytical parameters for the determination of MP at GNRs–Ag film modified SPCE with previously reported works.

Electrode	Linear range/μM	LOD/nM	Electrolyte	Methods	Ref.
MWCNTs–PAAM	0.005–10	2	PB buffer (pH 7.0)	DPV	19
Nano–TiO_2_/graphene composite	0.002–5 5–100	1	Acetate buffer (pH 5.2)	LSV	37
MIP–ionic liquid–graphene	0.010–7.0	6	PB buffer (pH 6.8)	DPV	16
ordered mesoporous carbon	0.09–61	7.6	PB buffer (pH 6.0)	LSV	18
carbon paste electrode	1–60	50	Acetate buffer (pH 5.2)	SWASV	38
Au nanoparticles/nafion	0.5–120	0.1	PB buffer (pH 7)	SWV	17
SH-β-CD/AuNPs/SWCNTs	0.002–0.080	0.1	PB buffer (pH 7.0)	SWV	39
Ag/MPTMS/OHP	0.0025–75	0.07	PB (pH 7)	DPV	40
Graphene/GdHCF	0.008–10	1	PB buffer (pH 6.0)	DPV	41
Ag/nafion composite	0.2–1.00	0.2815	B-R buffer (pH 2.56)	Amperometry	1
Ag@GNRs	0.005–2780	0.5	PB (pH 7)	Amperometry	This work

MWCNTs–PAAM = multiwalled carbon nanotubes–poly(acrylamide) nanocomposite; MIP- Molecularly imprinted polymer; SH-β-CD/AuNPs/SWCNTs = mono-6-thio-β-cyclodextrin self-assembled monolayer/gold nanoparticles/single-walled carbon nanotubes; Ag/MPTMS/OHP = Silver/3-mercaptopropyltrimethoxysilane/overhead projector; GdHCF = gadolinium hexacyanoferrate nanocomposite; PB buffer = phosphate buffer; B-R buffer = Britton-Robinson buffer; DPV = Differential pulse voltammetry; LSV = Linear sweep voltammetry; SWASV = square wave adsorptive stripping voltammetry; SWV = Square wave voltammetry.
